# MiR-708 inhibits MC3T3-E1 cells against H_2_O_2_-induced apoptosis through targeting PTEN

**DOI:** 10.1186/s13018-020-01780-w

**Published:** 2020-07-10

**Authors:** Wei Zhang, Sheng-Yu Cui, Hong Yi, Xin-Hui Zhu, Wei Liu, You-Jia Xu

**Affiliations:** 1 Department of Orthopedics, The Second Affiliated Hospital of Soochow University, 1055 Sanxiang Road, Suzhou, 215004 Jiangsu China; 2 Department of Orthopedics, The First People’s Hospital of Nantong, Nantong, 226001 Jiangsu Province China

**Keywords:** MiR-708, Apoptosis, MC3T3-E1, Reactive oxygen species, Osteogenesis

## Abstract

**Background:**

The dysregulation of proliferation and apoptosis plays a significant role in the pathogenesis of postmenopausal osteoporosis (PO). MicroRNAs play an important role in regulating apoptosis of MC3T3-E1 cells. However, the role and potential mechanism of miR-708 for regulating H_2_O_2_-induced apoptosis is unknown. This study aimed to investigate the protective function of miR-708 in H_2_O_2_-induced apoptosis of MC3T3-E1 osteoblasts.

**Methods:**

MC3T3-E1 was co-cultured with H_2_O_2_ for 8 h, then, flow cytometry, malondialdehyde (MDA), and glutathione peroxidase (Gpx) levels were measured to establish the oxidative model. MiRNA microarray was performed to assess differentially expressed miRNAs between control and H_2_O_2_-treated MC3T3-E1 cells. We then performed RT-PCR to identify the relative expression of miR-708 and PTEN. After transfected MC3T3-E1 with miR-708 mimics, flow cytometry, MDA, and Gpx level were performed to identify the apoptosis rate and oxidative stress in these groups. Furthermore, we small interfering RNA of PTEN to identify the role of PTEN in H_2_O_2_-induced apoptosis of MC3T3-E1 cells.

**Results:**

H_2_O_2_ (100 nM) could significantly induce the apoptosis of MC3T3-E1 cells. Moreover, H_2_O_2_ could significantly increase the MDA level and downregulated Gpx level. RT-PCR found that H_2_O_2_ significantly decrease the level of miR-708. Compared with H_2_O_2_ group, H_2_O_2_ + miR-708 mimic significantly decreased the apoptosis rate.

**Conclusions:**

miR-708 plays a protective role in H_2_O_2_-induced MC3T3-E1 osteoblasts apoptosis and its protective effect is proceeded by regulating ROS level and PTEN expression level.

## Introduction

Postmenopausal osteoporosis (PO) is one of the most common bone diseases, characterized by low bone mineral density (BMD) and osteoporotic fracture with high morbidity and mortality [1–3]. Currently, there are no ideal methods to cure osteoporotic fracture [4, 5]. Therefore, a comprehensive interpretation of the pathogenesis and molecular mechanism of osteoporosis is important to find the target marker of PO [6].

Bone remodeling is regulated by osteoblast and osteoclast. During bone metabolism, it is inevitably produced reactive oxygen species (ROS) [7]. Recent studies have found that ROS could induce osteoblast apoptosis and thus promote the progression of OP [8]. Moreover, ROS can significantly inhibit the osteogenic differentiation of bone marrow mesenchymal stem cells (BMSCs) [9].

microRNAs (miRNAs), one type of the non-coding RNAs, could regulate cellular ROS level and is closely related to OP. Lu et al. [10] revealed that miR-214 protects MC3T3-E1 osteoblasts against H_2_O_2_-induced apoptosis by suppressing oxidative stress and targeting ATF4. One of the microRNAs, miR-708, was found to have a negative role in regulating breast cancer [11] and osteosarcoma [12] metastasis. Another study revealed that miR-708 could directly targeting with Nrf2 in bovine granulosa cells, while Nrf2 is a redox-sensitive transcription factor regulating the expression of antioxidant genes [13]. Additionally, the abnormal expression of miR-708 has recently been associated with oxidative stress in neurodegenerative disorders [14].

In this report, we focused on the protective effect of miR-708 in H_2_O_2_-induced osteoblasts apoptosis and potential mechanism. We hypothesized that miR-708 inhibits MC3T3-E1 cells against H_2_O_2_-induced apoptosis through targeting PTEN.

## Chemicals and materials

H_2_O_2_ was purchased from Sigma-Aldrich (Aladdin, Shanghai, China). MC3T3-E1 cells were obtained from ATCC (Procell, Wuhan, China). Lipofectamine 3000 transfection reagent was obtained from Invitrogen (Carlsbad, CA, USA). Apoptosis assay kit was obtained from Keygen (Nanjing, Jiangsu, China). Malondialdehyde (MDA) and glutathione peroxidase (GPx) determination kits were obtained from Nanjing Jiancheng Biochemistry Co. (Nanjing, Jiangsu, China). β-actin and PTEN antibodies were purchased from Santa Cruz Biotechnology (Santa Cruz, CA, USA).

### Microarray hybridization

Total RNA was isolated from MC3T3-E1 cells and H_2_O_2_-treated MC3T3-E1 cells using TRIzol reagent (Invitrogen, USA) according to the manufacturer’s instructions. Microarray hybridization was performed by Agilent-070155 Mouse miRNA Microarray (miRBase Release 21.0, miRNA ID version) and performed by Cloud-seq Company (Shanghai, China). After normalization, differentially expressed miRNAs were performed by the Limma package of R software. Then, heatmap and volcano plot of the differentially expressed miRNAs were drawn using the pheatmap package of the R software.

### MC3T3-E1 cell culture

Murine osteoblastic MC3T3-E1 cells were maintained in a α-minimum essential medium (α-MEM) media supplement with 10% fetal bovine serum 100 U/ml penicillin at 37 °C in a humidified atmosphere of 5% CO_2_. MC3T3-E1 cells were seeded in a 6-well plate and divided into two groups: control group and H_2_O_2_ group. The concentration of H_2_O_2_ was 100 mM according to a previous report [15]. And we treated with 100 mM H_2_O_2_ for 8 h to induce the oxidative stress.

### MC3T3-E1 cell transfection

MC3T3-E1 cells (3 × 10^4^ cells/wells) were seeded in 24-well plates and incubated overnight. Transfection of the, miR-708, agomir-miR-708, antagomir-miR-708, corresponding negative control (NC), and si-PTEN was taken using Lipofectamine 3000 transfection reagent (Invitrogen). At 6 h posttransfection, the transfection medium was replaced by a regular culture medium containing 100 mM H_2_O_2_ for another 8 h.

### RNA isolation and real-time PCR

Total RNA was isolated by Trizol reagent (Invitrogen, Thermo Fisher, USA) according to the manufacturers’ instruction. RNA was analyzed by NanoDrop-2000 (Thermo Fisher, USA) for RNA quantification and purity. The cDNA was synthesized using the PrimeScript RT Master Mix (Takara, Japan) according to the manufacturer’s protocol. Synthesized cDNA was subsequently analyzed via qPCR using a SYBR Premix Ex TaqTM II (TliRNaseH Plus) kit (cat. no. RR820a; Takara Bio, Inc.). β-actin was used as an internal reference and the relative mRNA expression of target genes was calculated using the 2^−△△ct^ method. Primer sequence can be seen in Table 1.

**Table 1:** Primer of the sequence

Gene	Sequence
GAPDH	F: 5′-GGAGCGAGATCCCTCCAAAAT-3′
	R: 5′-GGCTGTTGTCATACTTCTCATGG-3′
U6	F: 5′-GCTTCGGCAGCACATATACTAAAAT-3′
	R: 5′-CGCTTCACGAATTTGCGTGTCAT-3′
miR-708	F: 5′-GGCGCGCAAGGAGCTTACAATC-3′
	R:5′-GTGCAGGGTCCGAGGTAT-3′
PTEN	F: 5′-ATTCCCAGTCAGAGGCGCTAT-3′
	R: 5′- GAACTTGTCTTCCCGTCGTGT-3′

### Western blot assay

Total proteins were isolated by using RIPA and PMSF (100:1). Same concentration of proteins was transferred to sodium dodecyl sulfate (SDS)-polyacrylamide gels. Subsequently, the protein was transferred in gel to polyvinylidene fluoride (PVDF) membranes. Then PVDF membrane was blocked by nonfat-dried milk and then incubated with primary antibodies at 4 °C overnight. PVDF membrane was washed with TBST and then incubated with secondary antibodies at room temperature for 1 h. Eventually, the electrochemical luminescence (ECL) solution was prepared in the darkroom. The exposure time was determined according to the fluorescence intensity.

### Cell apoptosis assay

MC3T3-E1 osteoblasts were seeded in a 6-well plate. MC3T3-E1 osteoblasts were divided into the following groups: control group, H_2_O_2_ (100 mM), miR-708 mimic, and H_2_O_2_ (100 mM) + miR-708 mimic. Apoptosis was examined by Annexin V-fluorescein isothiocyanate staining. Briefly, centrifuge and collection MC3T3-E1 osteoblasts, then MC3T3-E1 osteoblasts were washed by PBS for three times. Then, 5 μL of Annexin-V-FITC and 5 μL of pyridine iodide were added and incubated for 30 min. Finally, Becton-Dickinson FACS Caliber flow cytometer (BD Biosciences) was used to identify the apoptosis rate.

### ROS measurement

MC3T3-E1 cells in the above groups were collected by centrifuge. MDA and GPx levels were determined by MDA and GPx determination kits according to the manufacturer’s instruction. the concentration of MDA and the activity of GPx were detected by spectrophotometer at 580 nm.

### Luciferase reporter gene assay

Potential targets of miR-708 were predicted by performing a search in the following online database: miRanda (http://www.microrna.org/microrna/home.do) and TargetScan (http://www.targetscan.org/). 3′-UTR of the PTEN gene containing putative miR-708 targeting site was amplified by chemical synthesis and was inserted into the psiCHECK2 vector (Promega, Madison, WI, USA). When the confluence was up to 70%, MC3T3-E1 cells were transfected with related mixtures including 50 ng PTEN wild-type or PTEN mutant-type 3’-UTR reporter plasmids, miR-708 mimics or miR-708 NC in a final concentration of 20 nM, and Lipofectamine 3000 for 48 h. Luciferase activity was detected using the dual-luciferase reporter gene kit (Beyotime, Shanghai, China).

### Statistical analysis

All of the data are presented as means ± SD. Statistical analysis was performed using GraphPad Prism 7.00 (GraphPad Software, San Diego, USA). Student’s *t* test was used between two groups, while ANOVA followed by Dunnett’s test for multiple comparisons was conducted. A value of *p* < 0.05 was considered significant.

## Results

### Differentially expressed miRNAs

**Fig. 1:**
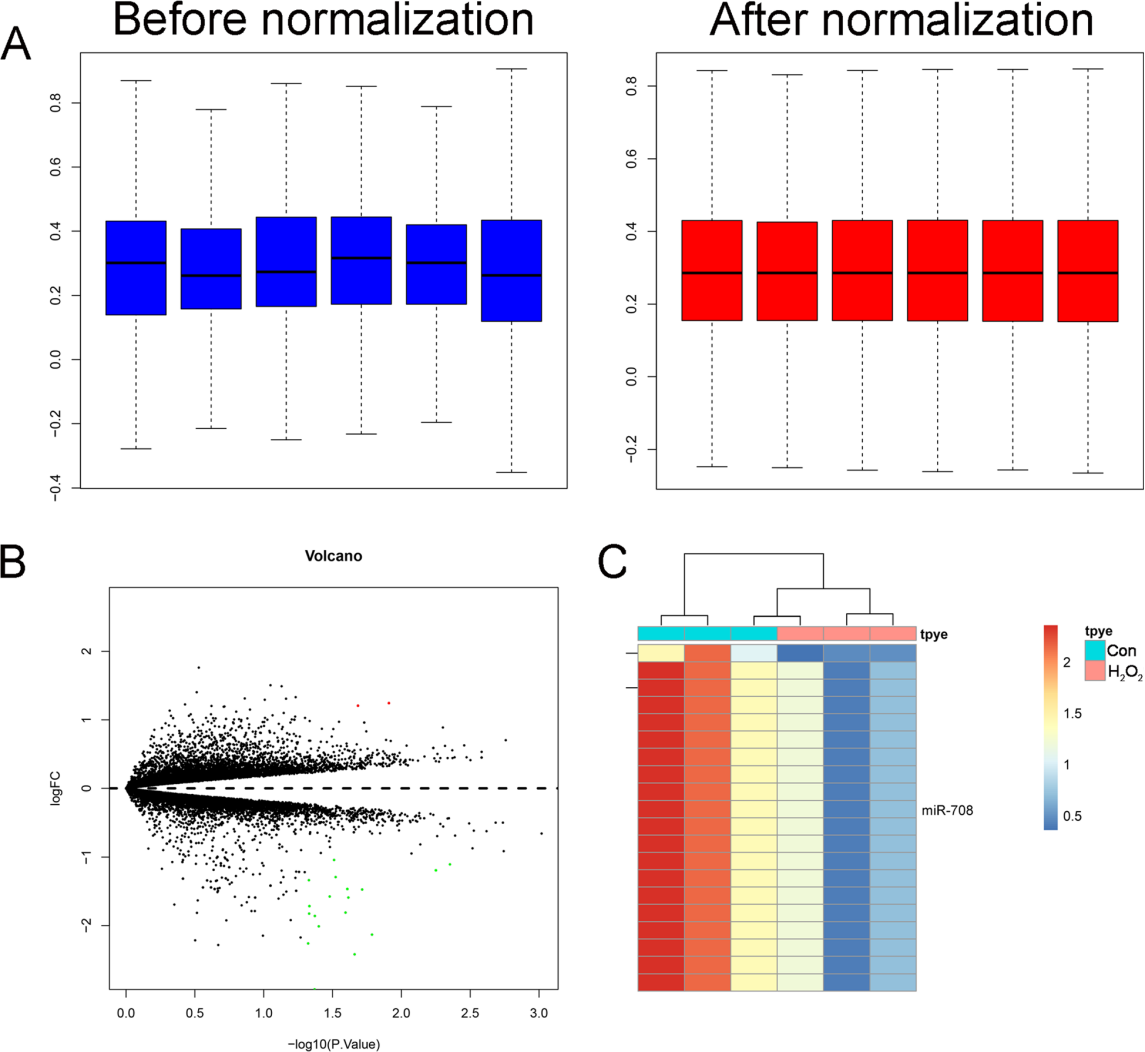
**a** Data normalization for differentially expressed miRNAs (data before normalization and after normalization. **b** Volcano plot the differentially expressed miRNAs. **c** Heatmap of the differentially expressed miRNAs

As shown in Fig. 1a and b, after data normalization, 74 miRNAs were identified, including 63 miRNAs and 11 miRNAs were downregulated and upregulated respectively (Fig. 1a and b). Volcano plot of the differentially expressed miRNAs can be seen in Fig. 1c. Heatmap of the differentially expressed miRNAs can be seen in Fig. 1d, and the miR-708 was the downregulated miRNA.

### H_2_O_2_-induced MC3T3-E1 apoptosis and elevated oxidative stress

**Fig. 2:**
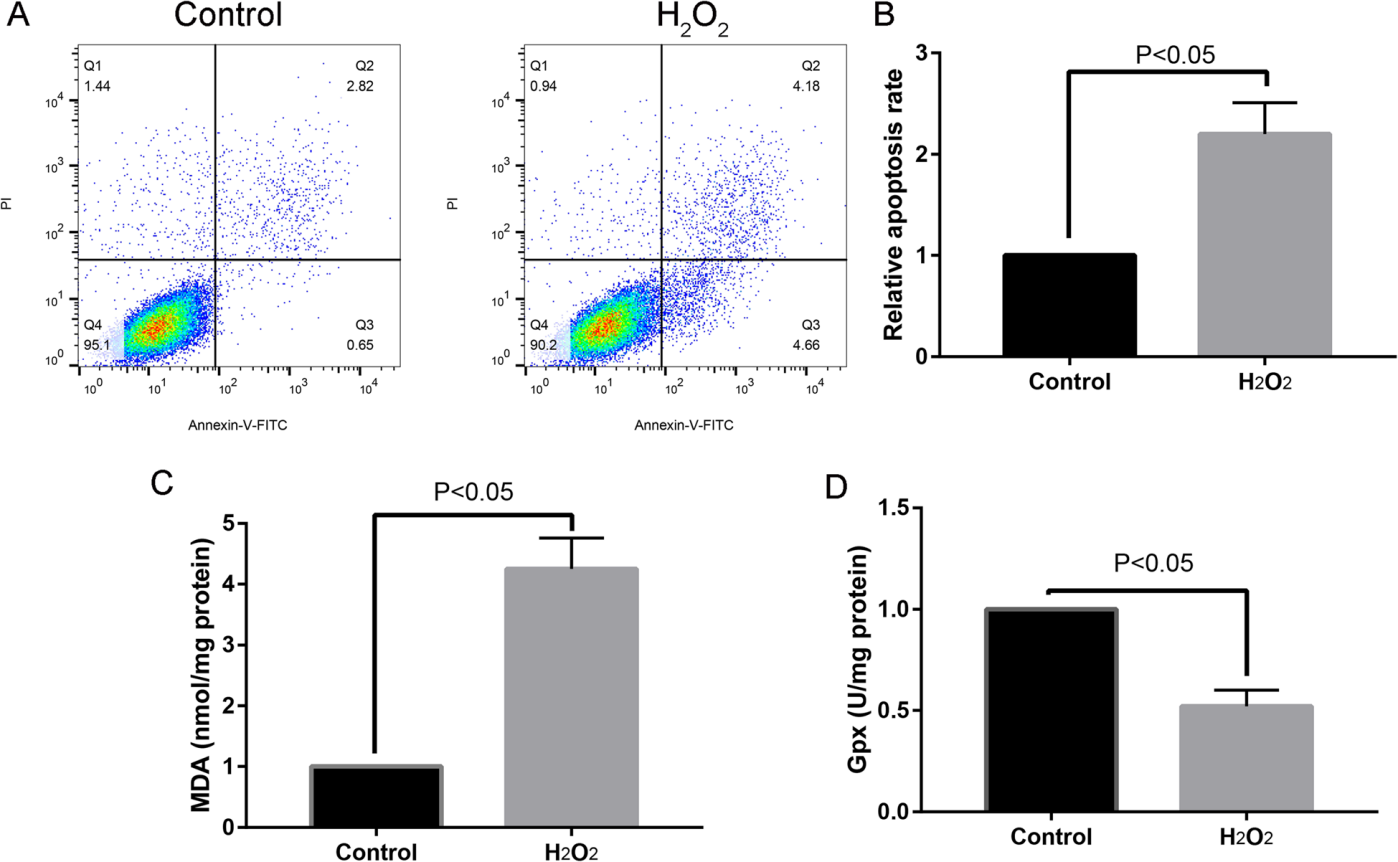
Apoptosis rate between H_2_O_2_ and control groups (**a** and **b**), MDA (**c**), and Gpx (**d**) level between H_2_O_2_ and control groups

After treatment with H_2_O_2_ to MC3T3-E1 cells for 24 h, MC3T3-E1 cells were harvested and performed Annexin-V-FITC analysis. Compared with the control group, adding H_2_O_2_ could significantly increase the apoptosis rate (Fig. 2a and b). Moreover, we measured the MDA and Gpx between the control and H_2_O_2_ groups. Results have shown that, compared with the control group, adding H_2_O_2_ could significantly increase the MDA (Fig. 2c, *P* < 0.05), while significantly decreased the Gpx level (Fig. 2d, *P* < 0.05).

### MiR-708 was decreased and PTEN was increased in H_2_O_2_-treated MC3T3-E1 cells

**Fig. 3:**
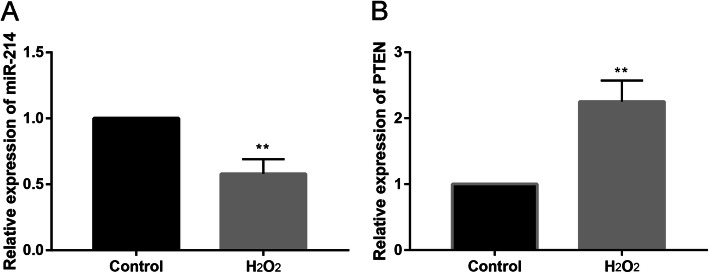
Relative expression of miR-708 and PTEN between H_2_O_2_ and control groups. **P < 0.05 compared with the control group

We further explored the miR-708 and PTEN expression between control and H_2_O_2_ groups. Compared with control group, H_2_O_2_ could significantly decrease the relative expression of miR-708 (Fig. 3a), while significantly increased the relative expression of PTEN (Fig. 3b).

### MiR-708 decreased H_2_O_2_-induced apoptosis and ROS level in MC3T3-E1 cells

**Fig. 4:**
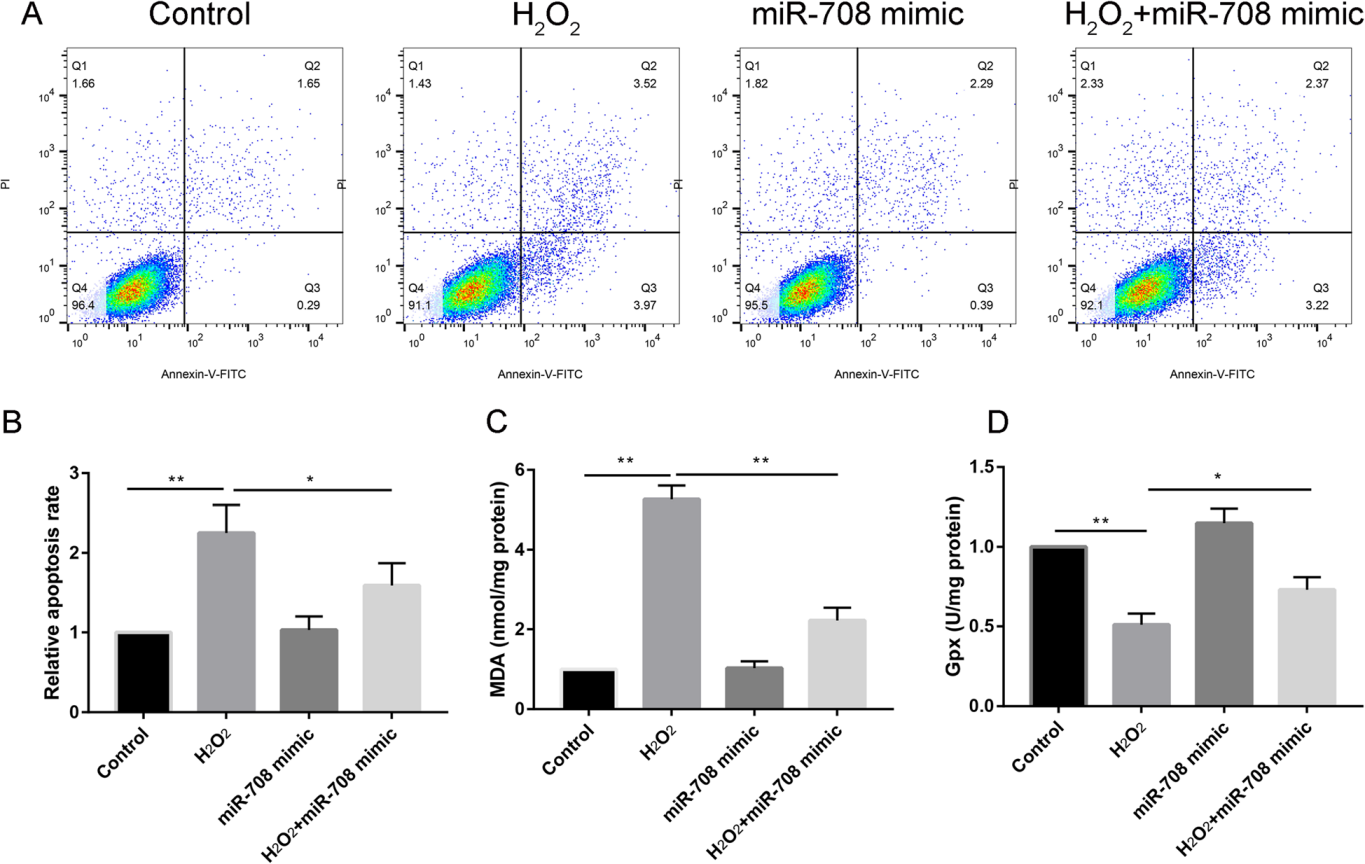
**a** The flow cytometry diagram for all groups. b The percentages of apoptotic cells for all groups. **c** MDA (**c**), and Gpx (**d**) level between H_2_O_2_, control group, miR-708 mimic, and H_2_O_2_ + miR-708 mimic groups

Compared with the control group, adding H_2_O_2_ significantly increased the apoptosis rate. There was no statistical difference between the miR-708 mimic and the control group in terms of the apoptosis rate (Fig. 4a and b). Compared with H_2_O_2_ alone, co-cultured H_2_O_2_ with miR-708 significantly decreased the apoptosis rate (*P* < 0.05). Compared with H_2_O_2_ group, extra adding miR-708 mimic could significantly decrease the MDA level (Fig. 4c) and increase the Gpx level (Fig. 4d, *P* < 0.05).

### PTEN is regulated by MiR-708

**Fig. 5:**
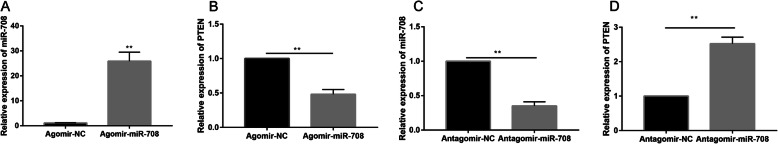
**a** Relative expression of miR-708 in agomir-NC and agomir-miR-708 groups. **b** Relative expression of PTEN in agomir-NC and agomir-miR-708 groups. **c** Relative expression of miR-708 (**c**) and PTEN (**d**) in antagomir-NC and antagomir-miR-708 groups

To further explore the relationship between miR-708 and PTEN, we used agomir-miR-708 and antagomir-miR-708 to explore the PTEN relative expression. Compared with agomir-NC, agomir-miR-708 could decrease the relative expression of PTEN (*P* < 0.05, Fig. 5). And when adding the antagomir-miR-708, the relative expression of miR-708 was significantly downregulated. And, compared with antagomir-NC, antagomir-miR-708 could significantly increase the relative expression of PTEN (Fig. 5).

### Inhibition the expression of PTEN reversed the apoptosis rate caused by H_2_O_2_

**Fig. 6:**
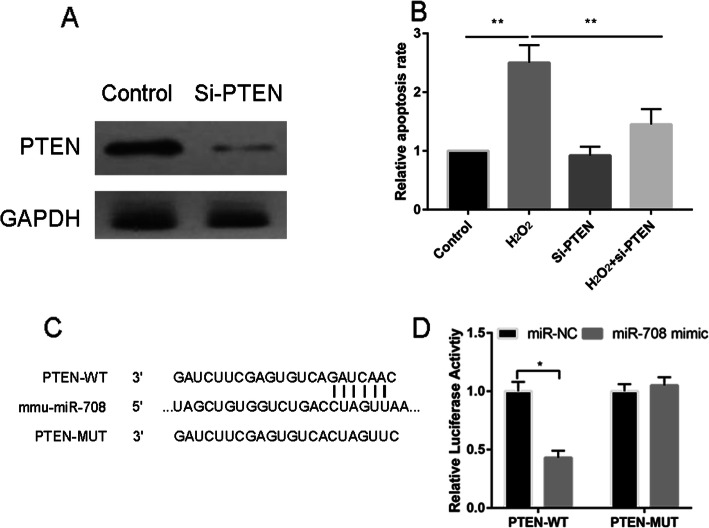
Western blot analyses of PTEN and cytochrome C control and si-PTEN groups.**b** Relative apoptosis rate in control, H_2_O_2,_ si-PTEN, and H_2_O_2+_ si-PTEN groups. **d** Luciferase reporter gene assay was conducted to further demonstrate the targeting relationship between PTEN and miR-708

Compared with the control group, si-PTEN could significantly decrease the protein expression of PTEN, which indicated that the si-PTEN could significantly downregulated the PTEN (Fig. 6a). Compared with the H_2_O_2_ group, extra adding si-PTEN could significantly decrease the apoptosis rate (Fig. 6b). Moreover, miR-708 could bind to the 576-582 of PTEN 3’UTR (Fig. 6c). The luciferase reporter gene assay in Fig. 6d further showed that the combination of PTEN-WT and miR-708 mimic largely decreased fluorescence intensity compared with the combination of PTEN-1-WT and miR-708 NC. However, the combination of PTEN-MUT and miR-708 mimic or miR-708 NC both have no effect on fluorescence intensity, indicating that there exists targeting relationship between PTEN and miR-708.

## Discussion

In our study, the inhibitory effect of miR-708 on MC3T3-E1 cells apoptosis was found to be mediated by the PTEN. Moreover, miR-708 mimic could also inhibit the oxidative damage caused by H_2_O_2_. This is the first study that explores the role of miR-708 in inhibiting H_2_O_2_-induced apoptosis. ROS are a significant pathogenic factor of osteoporosis. In this study, we used 100 μM H_2_O_2_ to build an oxidative damage model. Firstly, we measured the apoptosis rate, the level of MDA and Gpx, we found that H_2_O_2_ could increase the apoptosis rate and MDA level. And H_2_O_2_ could decrease the Gpx level than the control group.

Previous studies have found that oxidative stress could promote inhibit osteogenesis of bone marrow mesenchymal stem cells and promote the apoptosis of osteoblast [16]. Microarray hybridization was performed and found that miR-708 was downregulated in the H_2_O_2_ treated group. We found that miR-708 was decreased in the H_2_O_2_ group than the control group by PCR. Meanwhile, PTEN was the target gene of miR-708. Yang et al. [17] revealed that miR-21 promotes osteogenesis via the PTEN/PI3K/Akt/HIF-1α pathway and enhances bone regeneration in critical-size defects. PTEN has been implicated as an important regulator of osteoblast differentiation [18] and osteoblast apoptosis [19, 20]. And miR-17/PTEN axis could also promote osteoblasts viability [21]. We found that miR-708 could bind to the 576-582 of PTEN 3′ UTR and relative luciferase activity further identified that miR-708 could directly target with PTEN. Previously study found that PTEN is an inhibitor of the AKT signaling pathway and suppresses the expression of AKT [22]. Liu et al. [23] reported that PTEN modulates neuron apoptosis involving the PI3K/Akt/mTOR signaling pathway. Thus, PI3K/Akt is the downstream signaling pathway of PTEN.

MiR-708 possesses many physiological functions including regulating cell proliferation, apoptosis, and autophagy. Sun et al. [24] identified that miRNA-708 functions as a tumor suppressor in colorectal cancer by targeting ZEB1 through Akt/mTOR signaling pathway. Saini et al. [25] revealed that miR-708 induces apoptosis and suppresses tumorigenicity in renal cancer cells. In this study, we found that miR-708 could significantly reduce the H_2_O_2_-induced apoptosis of MC3T3-E1 cells.

## Conclusions

MiR-708 inhibits MC3T3-E1 osteoblasts against H_2_O_2_-induced apoptosis through directly targeting PTEN. Future studies should be focused on the effects of miR-708 for osteogenesis in vivo.

## Data Availability

We declare that the materials described in the manuscript will be freely available to all scientists for non-commercial purposes.

## Abbreviations

PO: Postmenopausal osteoporosis
MDA: Malondialdehyde
Gpx: Glutathione peroxidase
BMD: Bone mineral density
ROS: Reactive oxygen species
BMSCs: Bone marrow mesenchymal stem cells
miRNAs: microRNAs
αMEM: α-minimum essential medium
SDS: Sodium dodecyl sulfate
PVDF: Polyvinylidene fluoride
ECL: Electrochemical luminescence
